# Patterns of presentation of suspected and confirmed recurrent venous thromboembolism in patients with prior venous thromboembolism

**DOI:** 10.1016/j.rpth.2026.103442

**Published:** 2026-03-25

**Authors:** Vicky Mai, Emily S.L. Martens, Marc Righini, Sam Schulman, Venkatesh Thiruganasambandamoorthy, Veronica Bates, Amanda Pecarskie, Michael J. Kovacs, Shaun Visser, Sudeep Shivakumar, Melanie Tan, Marc Rodger, Dimitrios Scarvelis, Aurélien Delluc, Philippe Girard, Menno V. Huisman, Philip S. Wells, Frederikus A. Klok, Grégoire Le Gal, Susan R. Kahn, PREDICTORS study group, Marc Carrier, Catherine Code, Esteban Gandara, Lana Castellucci, Melissa Forgie, Carol Gonsalves, Lisa Duffett, Alan Karovitch, Timothy Ramsay, Mark Blostein, Vicky Tagalakis, Andrew Hirsch, Maral Koolian, Martha Louzada, Alejandro Lazo-Langner, Stephen Couban, David Anderson, Darrell White, David Macdonald, K. Sue Robinson, Wanda Hasegawa, Mary Margaret Keating, Matthew Mulligan, Marc Carrier, Kerstin de Wit, Lori Linkins, Alfonso Iorio, Jeff Weitz, Clive Kearon

**Affiliations:** 1 Centre de Recherche de l'Institut Universitaire de Cardiologie et de Pneumologie de Québec, Québec City, Québec, Canada; 2 Department of Medicine—Thrombosis and Hemostasis, Leiden University Medical Center, Leiden, the Netherlands; 3 Division of Angiology and Hemostasis, Geneva University Hospitals and Faculty of Medicine, Geneva, Switzerland; 4 Department of Medicine, Thrombosis and Atherosclerosis Research Institute, McMaster University, Hamilton, Ontario, Canada; 5 University of Ottawa, Department of Emergency Medicine, Ottawa, Ontario, Canada; 6 Department of Medicine, Ottawa Hospital Research Institute, University of Ottawa, Ottawa, Canada; 7 Division of Hematology, Department of Medicine, University of Western Ontario, London, Ontario, Canada; 8 Department of Emergency Medicine, Hôpital Montfort, Ottawa, Ontario, Canada; 9 Department of Medicine, Dalhousie University, Nova Scotia Health, Halifax, Nova Scotia, Canada; 10 Hospital Gelderse Vallei, Ede, the Netherlands; 11 Department of Medicine, McGill University, McGill University Health Center, Montreal, Québec, Canada; 12 Département de Pneumologie, Institut Mutualiste Montsouris, Paris, France; 13 French Clinical Research Infrastructure Network INNOVTE Network, Saint-Etienne, France; 14 Division of Internal Medicine, Department of Medicine, McGill University, Montreal, Québec, Canada

**Keywords:** deep venous thrombosis, pulmonary embolism, recurrence, venous thromboembolism

## Abstract

**Background:**

Patients with prior venous thromboembolism (VTE) seem to recur more frequently at the same site as their prior VTE, but their symptoms at presentation of suspected recurrent VTE is unclear.

**Objectives:**

The aim was to describe the patterns of presentation of suspected and confirmed recurrent VTE.

**Methods:**

This is a secondary analysis of the PREDICTORS study (ClinicalTrials.gov: NCT02297373), an international prospective multicenter observational cohort study. The primary outcomes were the location of symptoms and confirmed diagnosis of suspected recurrent VTE.

**Results:**

In total, 708 patients were included. Patients with isolated deep venous thrombosis (DVT) as the most recent prior VTE presenting with suspected recurrent VTE had more frequently lower extremity symptoms only (249/343; 73%), rather than respiratory symptoms only (41/343; 12%), and confirmed recurrent VTE were mostly isolated proximal DVT (78/100; 78%). Patients with isolated pulmonary embolism as the most recent prior VTE presented more frequently with respiratory symptoms only (136/237; 56%), rather than lower extremity symptoms only (42/237; 18%), and confirmed recurrent VTE were more frequently isolated pulmonary embolism (38/62; 61%). Among patients with DVT as the most recent prior VTE, confirmed recurrent DVT were more frequently in the ipsilateral leg of the index event (ipsilateral 72/468 [15%], contralateral 31/468 [7%]; odds ratio 2.3 [95% CI, 1.5-3.6]).

**Conclusion:**

In addition to confirming that patients were more frequently diagnosed with recurrent VTE at the same site of their last VTE, our study reported on suspected recurrent VTE symptoms and showed that patients presented more frequently with symptoms related to the same site of their last VTE.

## Essentials

• Symptoms at suspected recurrent VTE presentation in patients with prior VTE are unclear.
• This PREDICTORS secondary analysis describes patterns of suspected and confirmed recurrence.
• Recurrent VTE was diagnosed more often at the same site as the patient’s prior VTE.
• Patients more often presented with symptoms matching the site of their prior VTE.

## 1 Introduction

Approximately 15% to 20% of the patients who present with suspected venous thromboembolism (VTE) have experienced a prior venous thromboembolic event [[1], [2], [3], [4]]. Moreover, diagnosing a recurrent VTE can be challenging, especially when imaging after the initial anticoagulation period for the prior VTE is not available [5]. It is of high importance to adequately diagnose recurrent VTE since lifelong anticoagulation is usually recommended, which exposes the patient to an increase risk of bleeding [6].

The patterns of presentation of suspected and confirmed recurrent VTE are currently not well described. Patients with previous pulmonary embolism (PE) seem to have VTE recurrence more frequently as PE, and patients with prior deep venous thrombosis (DVT) seem to have VTE recurrence more frequently as DVT [[7], [8], [9], [10]]. However, it is not clear whether patients with prior VTE present more frequently with a suspicion of recurrent VTE at the same anatomical site (ie, lower extremity vs PE) as their last VTE event. Moreover, it is not clear whether patients with prior DVT present more frequently with a suspicion of DVT in the ipsilateral vs contralateral leg and whether the recurrent DVT is diagnosed more frequently in the same or opposite leg as the index event [11,12].

Better understanding of the patterns of presentation of suspected and diagnosed recurrent VTE will help clinicians in the diagnostic assessment of patients with prior VTE. To our knowledge, the side of symptom presentation of patients presenting with recurrent VTE had never been demonstrated in the past. The aim of this study was to describe the patterns of presentation of suspected and confirmed recurrent VTE in patients with prior VTE included in the PREDICTORS study.

## 2 Methods

This is a preplanned secondary analysis of the PREDICTORS study (ClinicalTrials.gov: NCT02297373), which was an international prospective multicenter observational cohort study of 723 patients presenting with suspected VTE recurrence at the emergency departments or thrombosis clinics at 6 centers in Canada, 1 center in Switzerland, and 1 center in the Netherlands [13]. The study was conducted from November 2014 to January 2019. Patients were included if they had signs or symptoms suggestive of recurrent VTE and were able to provide consent. Patients were excluded if it was unlikely that they would complete the 3 months follow-up due to their medical condition, if they presented with a suspicion of upper extremity thrombosis or thrombosis at an unusual site, if they had a distal DVT or subsegmental PE as their previous VTE, or if they had a diagnosis of upper extremity DVT or DVT from unknown site as their previous VTE. All VTE were adjudicated by an independent committee by using imaging reports. Only data collected at enrolment were used for this secondary analysis.

### 2.1 Outcomes

The primary outcomes were the patterns of presentation of suspected and confirmed recurrent VTE. Symptoms of recurrent VTE were lower extremity symptoms without respiratory symptoms, respiratory symptoms without lower extremity symptoms, and a combination of lower extremity and respiratory symptoms. Lower extremity symptoms were characterized by whole leg swelling, pain, cramps, itchiness, and/or heaviness. Respiratory symptoms were characterized by chest pain, dyspnea, and/or hemoptysis. Confirmed recurrent VTE were classified as isolated DVT, isolated PE, or DVT+PE at enrolment of this study. DVT were symptomatic proximal lower extremity DVT diagnosed by compression ultrasound (CUS) or magnetic resonance imaging, showing a new thrombus above the trifurcation of the popliteal vein. PE was diagnosed if a new unmatched segmental perfusion defect or greater was seen on a ventilation/perfusion (V/Q) scan, or if a new segmental or more proximal intraluminal defect was seen on a computed tomography pulmonary angiogram (CTPA). The side of the recurrent DVT was assessed during the entire study period and was ipsilateral, contralateral, bilateral, or unilateral when the last DVT occurred in both lower extremities.

### 2.2 Statistical analyses

Baseline characteristics are presented as mean ± SD if normally distributed for continuous variables and number (%) for dichotomous variables. For the patterns of presentation of suspected recurrent VTE, the proportion of patients with leg symptoms, respiratory symptoms, or both as symptoms leading to presentation for suspected recurrent VTE were calculated in the entire cohort and based on the type or anatomical location of the last VTE. For the patterns of presentation of confirmed recurrent VTE, the proportion of patients with confirmed isolated DVT, isolated PE, or DVT+PE were calculated in the entire cohort and based on the type or anatomical location of the last VTE. Among patients with the last VTE being DVT±PE, proportions of patients with suspected symptoms (respiratory, leg, or both) and confirmed recurrent VTE (isolated DVT, isolated PE, or DVT+PE) were calculated. Subgroup analyses were conducted based on anticoagulation status at time of inclusion (presence or absence), sex (female or male), residual thrombosis on CUS or residual pulmonary vascular obstruction (RPVO) on CTPA or V/Q scan (presence or absence), and postthrombotic syndrome (PTS; presence or absence, based on patient self-report). Sensitivity analysis was conducted based on the number of prior VTE events (1 vs ≥2 events) since the type or anatomical location of only the last VTE was known. Analyses were performed with SPSS (IBM Corp; released 2020; IBM SPSS Statistics for Macintosh, version 30.0.). Odds ratios (ORs) with its 95% CI were calculated when comparisons were made between 2 proportions. OR with its 95% CI were calculated using MedCalc Software Ltd (https://www.medcalc.org/en/calc/odds_ratio.php; version 23.4.8; accessed February 19, 2026).

## 3 Results

A total of 708 patients were included in this analysis since 15 patients were excluded as they had a suspicion of upper extremity DVT or a thrombosis from an unusual site. The mean age was 58 ± 17 years; 51% of the patients were female; 44% of the patients were on anticoagulation, mostly direct oral anticoagulant; and 16% were on antiplatelets. The location of last VTE was isolated DVT in 343 patients (48%), isolated PE in 237 patients (33%), and DVT+PE in 128 patients (18%). The last VTE was unprovoked in 470 patients (66%) (Table 1).

**Table 1:** Baseline characteristics of the patients.

Characteristics	Value (*N* = 708)
Age (y)	58 ± 17
Sex	
Female	363 (51)
Male	345 (49)
Race	
White	645 (91)
Black	28 (4)
Asian	19 (3)
Aboriginal	1 (0)
Hispanic	10 (1)
White and Black	2 (0)
White and Asian	1 (0)
White and Aboriginal	2 (0)
Body mass index (kg/m^2^)^a^	30 ± 7
Concomitant medication	
Antiplatelet	112 (16)
Anticoagulation	315 (44)
Oral estrogen therapy	16 (2)
Type of anticoagulation	
Warfarin	92 (13)
Direct oral anticoagulant	154 (22)
Low-molecular-weight heparin	62 (9)
Blinded investigational drug	7 (1)
Known thrombophilia	91 (13)
Family history of venous thromboembolism	210 (30)
No. of confirmed previous events	
1	552 (78)
≥2	156 (22)
Type of last venous thromboembolic event	
Pulmonary embolism	237 (33)
Deep venous thrombosis	343 (48)
Pulmonary embolism and deep venous thrombosis	128 (18)
Last event	
Provoked^b^	237 (34)
Unprovoked	470 (66)
Active cancer	77 (11)

Values are given as *n* (%) or mean ± SD.

a 702 participants.

b Provoked by surgery, trauma, hospitalization or malignancy-associated.

### 3.1 Patterns of presentation of suspected and confirmed recurrent VTE

Overall, 345 patients (49%) presented with lower extremity symptoms without respiratory symptoms, 217 patients (31%) presented with respiratory symptoms without lower extremity symptoms and 117 patients (17%) had both lower extremity and respiratory symptoms. Moreover, 106 patients (15%) were diagnosed with an isolated DVT, 72 patients (10%) with an isolated PE, and 25 patients (4%) with a concomitant diagnosis of DVT+PE. Among patients with an isolated DVT as their last VTE, these patients presented more frequently with lower extremity symptoms and were diagnosed more frequently with an isolated DVT. Among patients with isolated PE as their last VTE, these patients presented more frequently with respiratory symptoms and were more frequently diagnosed with isolated PE. Among patients with DVT+PE as their last VTE, these patients presented slightly more frequently with lower extremity symptoms and were diagnosed slightly more frequently with isolated PE (Table 2 and Figure A,B). Patients with DVT±PE as last VTE were more frequently diagnosed with a recurrent DVT in the ipsilateral lower extremity (72/468; 15%) compared with the contralateral lower extremity (31/468; 7%; OR. 2.3; 95 CI, 1.5-3.6) (Table 3). Moreover, there was no statistically significant difference in the incidence of recurrent VTE in patients presenting with symptoms in a different location from their last VTE compared with patients presenting with symptoms related to the same site of their last VTE (31% vs 27%; OR, 1.6; 95% CI, 0.8-2.1).

**Table 2:** Patterns of presentation of suspected and confirmed recurrent VTE at enrolment in the entire cohort (*N* = 708).

	Suspected recurrent VTE symptoms			Confirmed recurrent VTE		
	Lower extremity symptoms without respiratory symptoms	Respiratory symptoms without lower extremity symptoms	Lower extremity and respiratory symptoms	Isolated DVT	Isolated PE	DVT+PE
All patients (*N* = 708)	345 (49)	217 (31)	117 (17)	106 (15)	72 (10)	25 (4)
Patients with isolated DVT as last VTE (*n* = 343)	249 (73)	41 (12)	39 (11)	78 (23)	16 (5)	6 (2)
Patients with isolated PE as last VTE (*n* = 237)	42 (18)	136 (57)	54 (23)	14 (6)	38 (16)	10 (4)
Patients with PE+DVT as last VTE (*n* = 128)	54 (42)	40 (31)	24 (19)	14 (11)	18 (14)	9 (7)

Values are given as *n* (%).

DVT, deep venous thrombosis; PE, pulmonary embolism; VTE, venous thromboembolism.

**Figure:**
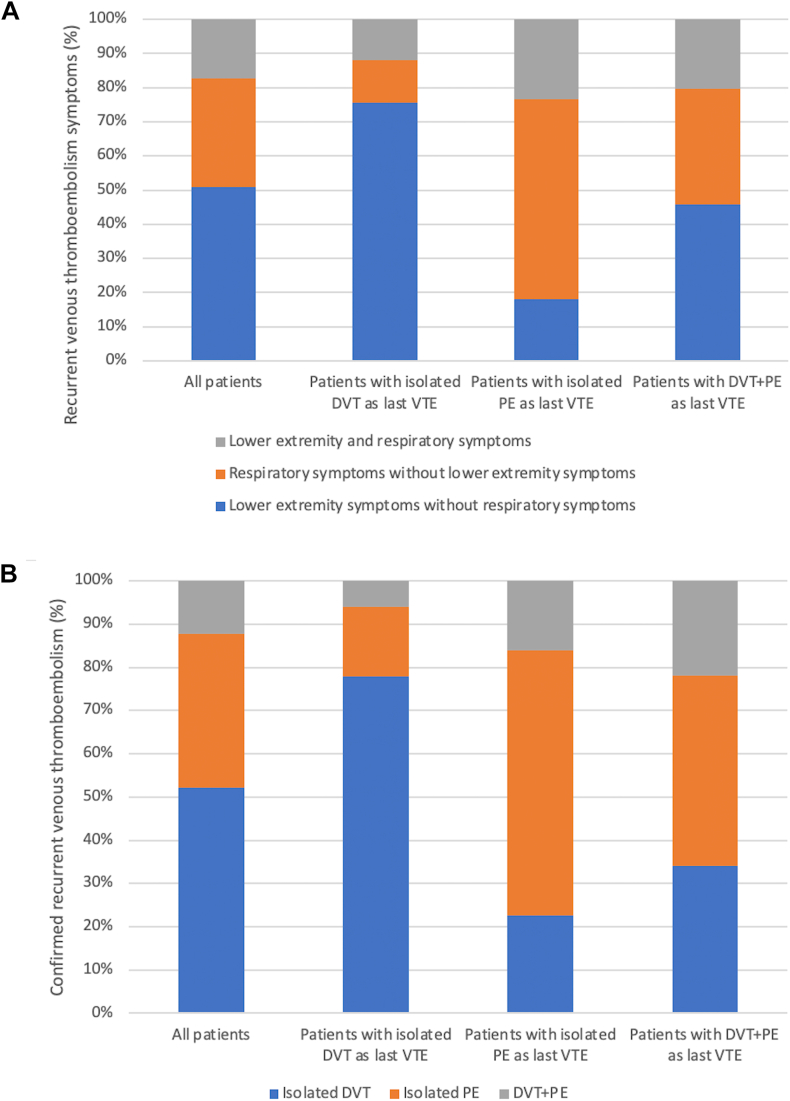
Patterns of presentation of suspected (A) and confirmed (B) recurrent venous thromboembolism (VTE) at enrolment in the entire cohort. DVT, deep vein thrombosis; PE, pulmonary embolism.

**Table 3:** Patterns of presentation of confirmed recurrent DVT in patients with DVT as last VTE.

	Confirmed recurrent DVT			
	Ipsilateral lower extremity	Contralateral lower extremity	Bilateral lower extremities	Unilateral when last DVT occurred in both lower extremities
Patients with DVT±PE as last VTE (*n* = 468^a^)	72 (15)	31 (7)	0 (0)	6 (1)
Patients with isolated DVT as last VTE (*n* = 341^b^)	55 (16)	26 (8)	0 (0)	4 (1)

Values are given as *n* (%).

DVT, deep venous thrombosis; PE, pulmonary embolism; VTE, venous thromboembolism.

a 3 patients were excluded from this analysis due to unknown side of the last lower extremity DVT.

b 2 patients were excluded from this analysis due to unknown side of the last lower extremity DVT.

### 3.2 Subgroup analyses

Subgroups analyses based on anticoagulation status at time of inclusion (Supplementary Tables 1 and 2), sex (Supplementary Tables 3 and 4), residual thrombosis on CUS or RPVO on CTPA or V/Q scan (Supplementary Tables 5 and 6), PTS (Supplementary Tables 7 and 8), and number of prior VTE (Supplementary Tables 9 and 10) showed mostly similar results in regard to the patterns of presentation of suspected and diagnosed recurrent VTE. However, the risk of recurrent VTE was higher in patients without anticoagulation compared to those on anticoagulation (41% vs 14%; OR 4.3; 95%CI 3.0-6.4) (Supplementary Table 1) and in males compared to females (34% vs 24%; OR 1.6; 95%CI 1.2-2.2) (Supplementary Table 3).

## 4 Discussion

Among 708 patients with prior VTE who presented with suspected recurrent VTE, our study showed for the first time to our knowledge that patients presented more frequently with symptoms related to the same site of their last VTE and also confirmed that these patients were more frequently diagnosed with recurrent VTE at the site of their last VTE. Moreover, patients with prior DVT were more likely to be diagnosed with a recurrence in the ipsilateral leg of the index event.

In the context of suspected recurrent VTE, our study showed that patients whose previous event was isolated DVT more often presented with symptoms confined to the lower extremities and were more frequently diagnosed with recurrent isolated DVT. Those whose previous event was a PE presented more frequently with respiratory symptoms only and were diagnosed more frequently with recurrent isolated PE. Patients with DVT+PE as last VTE presented slightly more frequently with lower extremity symptoms only but were diagnosed slightly more often with recurrent isolated PE. It makes sense that patients with isolated DVT or isolated PE as last VTE event are more susceptible to having residual thrombus in the lower extremity or RPVO, respectively. Residual thrombus or RPVO are known to predispose the patient to developing PTS and post-PE syndrome, respectively, which can mimic signs or symptoms of recurrent VTE [14,15]. The fact that recurrent VTE was more frequently diagnosed at the site of the index event is concordant with prior studies [[7], [8], [9], [10]] and may be explained by persistent inflammation created by the initial clot, as well as residual thrombus and prior injury to the vessel and its valves, which could lead to thrombosis [[16], [17], [18], [19]]. Residual thrombus in the lower extremity or RPVO are associated with increased risk of VTE recurrence [20,21]. Diagnostic bias may also partly explain these findings since patients presenting with suspected recurrent VTE present more frequently with symptoms related to the site of the last VTE, and consequently, the site is more frequently imaged. On the contrary, some could argue that those who present with symptoms in a different site of their prior VTE could have a higher incidence of recurrent VTE since those who have symptoms at the same site of their prior VTE might have their symptoms mostly explained by PTS or post-PE syndrome. However, our study was not able to confirm this difference since the incidence of recurrent VTE was similar in those 2 groups.

For patients with DVT as last VTE, recurrence was more frequently in the ipsilateral lower extremity than in the contralateral lower extremity. In the literature, data on the topic are unclear. One study showed that recurrence more frequently occurs in the contralateral leg, whereas another study demonstrated the recurrence occurs more commonly in the same leg [11,12] . It would make sense that recurrent DVT occur more frequently in the ipsilateral leg since up to 50% of the patients with prior DVT have residual vein thrombosis. Indeed, prior DVT increases local inflammatory processes and could have damaged venous valves, which can impair normal blood flow and predispose patients to a new VTE [16,22,23].

Subgroup analyses showed that the risk of recurrence was higher in males compared with females. This is in line with the literature showing that the annual risk of recurrent VTE in men was higher than in women among patients with unprovoked VTE who discontinued anticoagulation [24,25]. Subgroup analyses also showed that the risk of recurrence was higher in patients without anticoagulation than those with anticoagulation. These findings support the results from the PADIS-PE trial, where recurrent VTE occurred more often after anticoagulation was stopped [26].

We acknowledge that our study has some strengths. To our knowledge, this is the first study reporting on the symptoms of suspected recurrent VTE. Additionally, all recurrent VTE were adjudicated by an independent committee, which reduces the risk of diagnostic bias and increases the confidence we have in the results, especially in patients with recurrent VTE where the diagnosis may be challenging. Moreover, with a similar incidence of recurrent VTE in patients presenting with symptoms in a different location from their last VTE and in patients presenting with symptoms related to the same site of their last VTE, the risk of misclassification is unlikely. We acknowledge that our study also presents some limitations. First, some subgroup analyses comprised only a small number of patients, which could reflect some results to be viewed as exploratory. Second, among those with suspected recurrent DVT, the side (ipsilateral or contralateral) of the symptoms was not available for all patients, which limited the information we could have on the clinical patterns of presentation of suspected recurrent DVT. Third, while subgroup analysis on PTS was performed, the presence or absence of PTS was based on the information reported by the patient, which could bring some misclassification.

## 5 Conclusion

In addition to confirming that patients were more frequently diagnosed with recurrent VTE at the same site of their index VTE, our study reported on suspected recurrent VTE symptoms and showed that patients presented more frequently with symptoms related to the same site of their index VTE among all patients presenting with a suspicion of recurrent VTE. Patients with prior DVT were more likely to present with symptoms and be diagnosed with recurrence in the ipsilateral leg of the index event. Better understanding of the patterns of presentation of suspected recurrent VTE could improve diagnostic investigations.

## Appendices

Members of PREDICTORS study group

**Ottawa (Ottawa Hospital), Ontario, Canada**: Marc Carrier, Catherine Code, Esteban Gandara, Lana Castellucci, Melissa Forgie, Carol Gonsalves, Lisa Duffett, Alan Karovitch, Timothy Ramsay.

**Montreal, Quebec, Canada**: Mark Blostein, Vicky Tagalakis, Andrew Hirsch, Maral Koolian.

**London, Ontario, Canada**: Martha Louzada, Alejandro Lazo-Langner.

**Halifax, Nova Scotia, Canada**: Stephen Couban, David Anderson, Darrell White, David Macdonald, K. Sue Robinson, Wanda Hasegawa, Mary Margaret Keating.

**Ottawa (Montfort Hospital), Ontario, Canada**: Matthew Mulligan, Marc Carrier.

**Hamilton, Ontario, Canada**: Kerstin de Wit, Lori Linkins, Alfonso Iorio, Jeff Weitz, Clive Kearon.
