# Supplementary material for: Patterns of presentation of suspected and confirmed recurrent venous thromboembolism in patients with prior venous thromboembolism
Source: Res Pract Thromb Haemost. 2026 Mar 25;10(3):103442. doi: 10.1016/j.rpth.2026.103442 (PMC13122812; doi:10.1016/j.rpth.2026.103442)
Supplement: Supplementary Tables [file mmc1.docx]

**Patterns of presentation of suspected and confirmed recurrent venous thromboembolism in patients with prior venous thromboembolism**

**Supplement**

Vicky Mai^1^, Emily S. Martens^2^, Marc Righini^3^, Sam Schulman^4^, Venkatesh Thiruganasambandamoorthy^5^, Veronica Bates^1^, Amanda Pecarskie^1^, Michael J. Kovacs^6^, Shaun Visser^7^, Sudeep Shivakumar^8^, Melanie Tan^9^, Marc Rodger^10^, Dimitrios Scarvelis^1^, Aurélien Delluc^1^, Philippe Girard^11^, Menno V. Huisman^2^, Philip S. Wells^1^, Frederikus A. Klok^2^, Grégoire Le Gal^1^, Susan R. Kahn^12^– for the PREDICTORS study group.

^1^ Department of Medicine, Ottawa Hospital Research Institute, University of Ottawa, Ottawa, Canada.

^2^ Department of Medicine - Thrombosis and Hemostasis, Leiden University Medical Center, Leiden, the Netherlands.

^3^ Division of Angiology and Hemostasis, Geneva University Hospitals and Faculty of Medicine, Geneva, Switzerland.

^4^ Department of Medicine, Thrombosis and Atherosclerosis Research Institute, McMaster University, Hamilton, Ontario, Canada.

^5^ University of Ottawa, Department of Emergency Medicine, Ottawa, Ontario, Canada.

^6^ Division of Hematology, Department of Medicine, University of Western Ontario, London, Ontario, Canada.

^7^ Department of Emergency Medicine, Hôpital Montfort, Ottawa, Ontario, Canada.

^8^ Department of Medicine, Dalhousie University, Nova Scotia Health, Halifax, Nova Scotia, Canada.

^9^ Amsterdam University Medical Center, Amsterdam, Netherlands.

^10^ Department of Medicine, McGill University, McGill University Health Center, Montreal, Quebec, Canada.

^11^ Département de Pneumologie, Institut Mutualiste Montsouris, Paris, France; F-CRIN INNOVTE Network, Saint-Etienne, France.

^12^ Division of Internal Medicine, Department of Medicine, McGill University, Montreal, Québec, Canada.

**Supplementary Table 1. Patterns of presentation of suspected and confirmed recurrent venous thromboembolism at enrollment according to the anticoagulation status**

|  | Suspected recurrent VTE symptoms | | | Confirmed recurrent VTE | | |
| --- | --- | --- | --- | --- | --- | --- |
|  | Lower extremity symptoms without respiratory symptoms  n (%) | Respiratory symptoms without lower extremity symptoms  n (%) | Lower extremity and respiratory symptoms  n (%) | Isolated DVT  n (%) | Isolated PE  n (%) | DVT+PE  n (%) |
| *No anticoagulation (n=393)* | | | | | | |
| All patients (n=393) | 187 (48) | 130 (33) | 65 (17) | 77 (20) | 59 (15) | 24 (6) |
| Patients with isolated DVT as last VTE (n=192) | 140 (73) | 26 (14) | 20 (10) | 59 (31) | 11 (6) | 5 (3) |
| Patients with isolated PE as last VTE (n=133) | 19 (14) | 80 (60) | 31 (23) | 10 (8) | 33 (25) | 10 (8) |
| Patients with PE+DVT as last VTE (n=68) | 28 (41) | 24 (35) | 14 (21) | 8 (12) | 15 (22) | 9 (13) |
| *Anticoagulation (n=315)* | | | | | | |
| All patients (n=315) | 158 (50) | 87 (28) | 52 (17) | 29 (9) | 13 (4) | 1 (0) |
| Patients with isolated DVT as last VTE (n=151) | 109 (72) | 15 (10) | 19 (13) | 19 (13) | 5 (3) | 1 (1) |
| Patients with isolated PE as last VTE (n=104) | 23 (22) | 56 (54) | 23 (22) | 4 (4) | 5 (5) | 0 (0) |
| Patients with PE+DVT as last VTE (n=60) | 26 (43) | 16 (27) | 10 (17) | 6 (10) | 3 (5) | 0 (0) |

DVT: deep venous thrombosis; PE: pulmonary embolism; VTE: venous thromboembolism;

**Supplementary Table 2.** **Patterns of presentation of confirmed recurrent DVT in patients with DVT as last VTE according to the anticoagulation status**

|  | Confirmed recurrent DVT | | | |
| --- | --- | --- | --- | --- |
|  | Ipsilateral lower extremity  n (%) | Contralateral lower extremity  n (%) | Bilateral lower extremities  n (%) | Unilateral when last DVT occurred in both lower extremities n (%) |
| *No anticoagulation (n=258)* | | | | |
| Patients with DVT±PE as last VTE (n=258)^*^ | 54 (21) | 24 (9) | 0 (0) | 4 (2) |
| Patients with isolated DVT as last VTE (n=190)^*^ | 40 (21) | 21 (11) | 0 (0) | 4 (2) |
| *Anticoagulation (n=210)* | | | | |
| Patients with DVT±PE as last VTE (n=210)^α^ | 18 (9) | 7 (3) | 0 (0) | 2 (1) |
| Patients with isolated DVT as last VTE (n=151) | 15 (10) | 5 (3) | 0 (0) | 0 (0) |

^*^2 patients were excluded from this analysis due to unknown side of the last lower extremity DVT; ^α^1 patient was excluded from this analysis due to unknown side of the last lower extremity DVT; DVT: deep venous thrombosis; PE: pulmonary embolism; VTE: venous thromboembolism;

**Supplementary Table 3. Patterns of presentation of suspected and confirmed recurrent venous thromboembolism at enrollment according to sex**

|  | Suspected recurrent VTE symptoms | | | Confirmed recurrent VTE | | |
| --- | --- | --- | --- | --- | --- | --- |
|  | Lower extremity symptoms without respiratory symptoms  n (%) | Respiratory symptoms without lower extremity symptoms  n (%) | Lower extremity and respiratory symptoms  n (%) | Isolated DVT  n (%) | Isolated PE  n (%) | DVT+PE  n (%) |
| *Female (n=363)* | | | | | | |
| All patients (n=363) | 163 (45) | 114 (31) | 75 (21) | 39 (11) | 36 (10) | 12 (3) |
| Patients with isolated DVT as last VTE (n=165) | 118 (72) | 21 (13) | 21 (13) | 30 (18) | 8 (5) | 3 (2) |
| Patients with isolated PE as last VTE (n=131) | 17 (13) | 72 (55) | 40 (31) | 5 (4) | 22 (17) | 5 (4) |
| Patients with PE+DVT as last VTE (n=67) | 28 (42) | 21 (31) | 14 (21) | 4 (6) | 6 (9) | 4 (6) |
| *Male (n=345)* | | | | | | |
| All patients (n=345) | 182 (53) | 103 (30) | 42 (12) | 67 (19) | 36 (10) | 13 (4) |
| Patients with isolated DVT as last VTE (n=178) | 131 (74) | 20 (11) | 18 (10) | 48 (27) | 8 (4) | 3 (2) |
| Patients with isolated PE as last VTE (n=106) | 25 (24) | 64 (60) | 14 (13) | 9 (8) | 16 (15) | 5 (5) |
| Patients with PE+DVT as last VTE (n=61) | 26 (43) | 19 (31) | 10 (16) | 10 (16) | 12 (20) | 5 (8) |

DVT: deep venous thrombosis; PE: pulmonary embolism; VTE: venous thromboembolism;

**Supplementary Table 4.** **Patterns of presentation of confirmed recurrent DVT in patients with DVT as last VTE according to sex**

|  | Confirmed recurrent DVT | | | |
| --- | --- | --- | --- | --- |
|  | Ipsilateral lower extremity  n (%) | Contralateral lower extremity  n (%) | Bilateral lower extremities  n (%) | Unilateral when last DVT occurred in both lower extremities n (%) |
| *Female (n=230)* | | | | |
| Patients with DVT±PE as last VTE (n=230)^*^ | 28 (12) | 12 (5) | 0 (0) | 3 (1) |
| Patients with isolated DVT as last VTE (n=163)^*^ | 22 (13) | 11 (7) | 0 (0) | 1 (1) |
| *Male (n=238)* | | | | |
| Patients with DVT±PE as last VTE (n=238)^α^ | 44 (18) | 19 (8) | 0 (0) | 3 (1) |
| Patients with isolated DVT as last VTE (n=178) | 33 (19) | 15 (8) | 0 (0) | 3 (2) |

^*^2 patients were excluded from this analysis due to unknown side of the last lower extremity DVT; ^α^1 patient was excluded from this analysis due to unknown side of the last lower extremity DVT; DVT: deep venous thrombosis; PE: pulmonary embolism; VTE: venous thromboembolism;

**Supplementary Table 5. Patterns of presentation of suspected and confirmed recurrent venous thromboembolism at enrollment according to presence of residual thrombosis on CUS or pulmonary vascular obstruction on CTPA or V/Q scan**

|  | Suspected recurrent VTE symptoms | | | Confirmed recurrent VTE | | |
| --- | --- | --- | --- | --- | --- | --- |
|  | Lower extremity symptoms without respiratory symptoms  n (%) | Respiratory symptoms without lower extremity symptoms  n (%) | Lower extremity and respiratory symptoms  n (%) | Isolated DVT  n (%) | Isolated PE  n (%) | DVT+PE  n (%) |
| *Absence of residual thrombosis (n=515)* | | | | | | |
| All patients (n=515) | 226 (44) | 175 (34) | 93 (18) | 72 (14) | 60 (12) | 19 (4) |
| Patients with isolated DVT as last VTE (n=229) | 161 (70) | 29 (13) | 30 (13) | 47 (21) | 14 (6) | 3 (1) |
| Patients with isolated PE as last VTE (n=201) | 33 (16) | 116 (58) | 47 (23) | 14 (7) | 32 (16) | 9 (4) |
| Patients with PE+DVT as last VTE (n=85) | 32 (38) | 30 (35) | 16 (19) | 11 (13) | 14 (16) | 7 (8) |
| *Presence of residual thrombosis (n=193)* | | | | | | |
| All patients (n=193) | 119 (62) | 42 (22) | 24 (12) | 34 (18) | 12 (6) | 6 (3) |
| Patients with isolated DVT as last VTE (n=114) | 88 (77) | 12 (11) | 9 (8) | 31 (27) | 2 (2) | 3 (3) |
| Patients with isolated PE as last VTE (n=36) | 9 (25) | 20 (56) | 7 (19) | 0 (0) | 6 (17) | 1 (3) |
| Patients with PE+DVT as last VTE (n=43) | 22 (51) | 10 (23) | 8 (19) | 3 (7) | 4 (9) | 2 (5) |

CTPA: computed tomography pulmonary angiogram; CUS: compression ultrasonography; DVT: deep venous thrombosis; PE: pulmonary embolism; V/Q: ventilation/perfusion; VTE: venous thromboembolism;

**Supplementary Table 6.** **Patterns of presentation of confirmed recurrent DVT in patients with DVT as last VTE according to presence of residual thrombosis on CUS or pulmonary vascular obstruction on CTPA or V/Q scan**

|  | Confirmed recurrent DVT | | | |
| --- | --- | --- | --- | --- |
|  | Ipsilateral lower extremity  n (%) | Contralateral lower extremity  n (%) | Bilateral lower extremities  n (%) | Unilateral when last DVT occurred in both lower extremities n (%) |
| *Absence of residual thrombosis (n=311)* | | | | |
| Patients with DVT±PE as last VTE (n=311)^*^ | 43 (14) | 20 (6) | 0 (0) | 6 (2) |
| Patients with isolated DVT as last VTE (n=227)^α^ | 30 (13) | 16 (7) | 0 (0) | 4 (2) |
| *Presence of residual thrombosis (n=157)* | | | | |
| Patients with DVT±PE as last VTE (n=157) | 29 (18) | 11 (7) | 0 (0) | 0 (0) |
| Patients with isolated DVT as last VTE (n=114) | 25 (22) | 10 (9) | 0 (0) | 0 (0) |

^*^3 patients were excluded from this analysis due to unknown side of the last lower extremity DVT; ^α^2 patients were excluded from this analysis due to unknown side of the last lower extremity DVT; CTPA: computed tomography pulmonary angiogram; CUS: compression ultrasonography; DVT: deep venous thrombosis; PE: pulmonary embolism; V/Q: ventilation/perfusion; VTE: venous thromboembolism;

**Supplementary Table 7. Patterns of presentation of suspected and confirmed recurrent venous thromboembolism at enrollment according to presence of post-thrombotic syndrome among patients who had a deep venous thrombosis as last venous thromboembolism**

|  | Suspected recurrent VTE symptoms | | | Confirmed recurrent VTE | | |
| --- | --- | --- | --- | --- | --- | --- |
|  | Lower extremity symptoms without respiratory symptoms  n (%) | Respiratory symptoms without lower extremity symptoms  n (%) | Respiratory and lower extremity symptoms  n (%) | Isolated DVT  n (%) | Isolated PE  n (%) | DVT+PE  n (%) |
| *Absence of post-thrombotic syndrome (n=266)* | | | | | | |
| All patients (n=266) | 153 (58) | 59 (22) | 37 (14) | 52 (20) | 27 (10) | 9 (3) |
| Patients with isolated DVT as last VTE (n=182) | 128 (70) | 25 (14) | 20 (11) | 41 (23) | 13 (7) | 1 (1) |
| Patients with PE+DVT as last VTE (n=84) | 25 (30) | 34 (40) | 17 (20) | 11 (13) | 14 (17) | 8 (10) |
| *Presence of post-thrombotic syndrome (n=205)* | | | | | | |
| All patients (n=205) | 150 (73) | 22 (11) | 26 (13) | 40 (20) | 7 (3) | 6 (3) |
| Patients with isolated DVT as last VTE (n=161) | 121 (75) | 16 (10) | 19 (12) | 37 (23) | 3 (2) | 5 (3) |
| Patients with PE+DVT as last VTE (n=44) | 29 (66) | 6 (14) | 7 (16) | 3 (7) | 4 (10) | 1 (2) |

DVT: deep venous thrombosis; PE: pulmonary embolism; VTE: venous thromboembolism;

**Supplementary Table 8.** **Patterns of presentation of confirmed recurrent DVT in patients with DVT as last VTE according to presence of post-thrombotic syndrome among patients who had a deep venous thrombosis as last venous thromboembolism**

|  | Confirmed recurrent DVT | | | |
| --- | --- | --- | --- | --- |
|  | Ipsilateral lower extremity  n (%) | Contralateral lower extremity  n (%) | Bilateral lower extremities  n (%) | Unilateral when last DVT occurred in both lower extremities n (%) |
| *Absence of post-thrombotic syndrome (n=263)* | | | | |
| Patients with DVT±PE as last VTE (n=263)^*^ | 44 (17) | 15 (6) | 0 (0) | 3 (1) |
| Patients with isolated DVT as last VTE (n=180)^α^ | 29 (16) | 11 (6) | 0 (0) | 2 (1) |
| *Presence of post-thrombotic syndrome (n=205)* | | | | |
| Patients with DVT±PE as last VTE (n=205) | 28 (14) | 16 (8) | 0 (0) | 3 (1) |
| Patients with isolated DVT as last VTE (n=161) | 26 (16) | 15 (9) | 0 (0) | 2 (1) |

^*^3 patients were excluded from this analysis due to unknown side of the last lower extremity DVT; ^α^2 patients were excluded from this analysis due to unknown side of the last lower extremity DVT; DVT: deep venous thrombosis; PE: pulmonary embolism; VTE: venous thromboembolism;

**Supplementary Table 9. Patterns of presentation of suspected and confirmed recurrent venous thromboembolism in the entire cohort according to the number of prior venous thromboembolic events**

|  | Suspected recurrent VTE symptoms | | | Confirmed recurrent VTE | | |
| --- | --- | --- | --- | --- | --- | --- |
|  | Lower extremity symptoms without respiratory symptoms  n (%) | Respiratory symptoms without lower extremity symptoms  n (%) | Respiratory and lower extremity symptoms  n (%) | Isolated DVT  n (%) | Isolated PE  n (%) | DVT+PE  n (%) |
| *1 prior venous thromboembolic event (n=552)* | | | | | | |
| All patients (n=552) | 261 (47) | 168 (30) | 98 (18) | 78 (14) | 53 (10) | 22 (4) |
| Patients with isolated DVT as last VTE (n=262) | 190 (73) | 30 (11) | 31 (12) | 57 (22) | 12 (5) | 4 (2) |
| Patients with isolated PE as last VTE (n=185) | 27 (15) | 107 (58) | 47 (25) | 10 (5) | 30 (16) | 9 (5) |
| Patients with PE+DVT as last VTE (n=105) | 44 (42) | 31 (30) | 20 (19) | 11 (10) | 11 (10) | 9 (9) |
| *≥ 2 prior venous thromboembolic events (n-156)* | | | | | | |
| All patients (n=156) | 84 (54) | 49 (31) | 19 (12) | 28 (18) | 19 (12) | 3 (2) |
| Patients with isolated DVT as last VTE (n=81) | 59 (73) | 11 (14) | 8 (10) | 21 (26) | 4 (5) | 2 (2) |
| Patients with isolated PE as last VTE (n=52) | 15 (29) | 29 (56) | 7 (13) | 4 (8) | 8 (15) | 1 (2) |
| Patients with PE+DVT as last VTE (n=23) | 10 (43) | 9 (39) | 4 (17) | 3 (13) | 7 (30) | 0 (0) |

DVT: deep venous thrombosis; PE: pulmonary embolism; VTE: venous thromboembolism;

**Supplementary Table 10.** **Patterns of presentation of confirmed recurrent DVT in patients with DVT as last VTE according to the number of prior venous thromboembolic events**

|  | Confirmed recurrent DVT | | | |
| --- | --- | --- | --- | --- |
|  | Ipsilateral lower extremity  n (%) | Contralateral lower extremity  n (%) | Bilateral lower extremities  n (%) | Unilateral when last DVT occurred in both lower extremities n (%) |
| *1 prior venous thromboembolic event (n=364)* | | | | |
| Patients with DVT±PE as last VTE (n=364)^*^ | 53 (15) | 26 (7) | 0 (0) | 4 (1) |
| Patients with isolated DVT as last VTE (n=260)^α^ | 38 (15) | 22 (8) | 0 (0) | 2 (1) |
| *≥ 2 prior venous thromboembolic events (n=104)* | | | | |
| Patients with DVT±PE as last VTE (n=104) | 19 (18) | 5 (5) | 0 (0) | 2 (2) |
| Patients with isolated DVT as last VTE (n=81) | 17 (21) | 4 (5) | 0 (0) | 2 (2) |

^*^3 patients were excluded from this analysis due to unknown side of the last lower extremity DVT; ^α^2 patients were excluded from this analysis due to unknown side of the last lower extremity DVT; DVT: deep venous thrombosis; PE: pulmonary embolism; VTE: venous thromboembolism;
